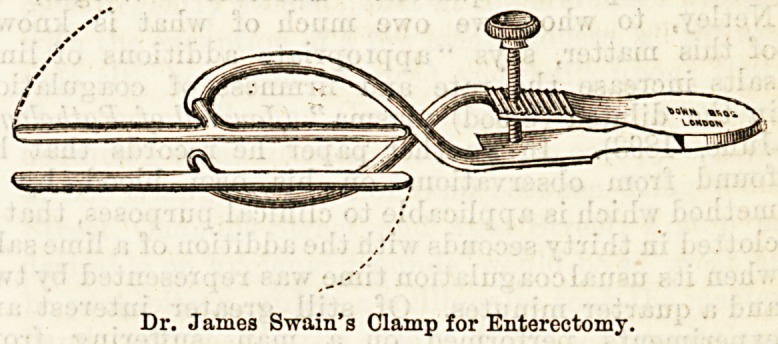# Surgery of Intestines

**Published:** 1894-02-03

**Authors:** 


### SURGERY OF INTESTINES.

The treatment of gangrenous hernia by means of resection and immediate suture of the intestine, in preference to the formation of an artificial anus, which requires a subsequent operation, is fast gaining ground, and has recently been advocated[1] by Mr. Kendal Franks, who quotes a successful case. Beck also reports[2] two successful cases. Mr. Franks says that, in order to estimate the relative merits of these two principles of treatment, the death-rate of immediate resection and suture must be compared with the death-rate following the formation of an artificial anus, plus the death-rate of the secondary operation required for its cure. The death-rate in cases of gangrenous hernia treated by the formation of an artificial anus was 80·7 per cent.

The death rate of secondary resection and suture for the cure of artificial anus was 38 per cent. The death-rate following the use of the enterotome was 7·3 per cent. The mortality which attended resection and immediate suture in gangrenous hernia was shown—in a table of 220 published cases—to be 48 per cent. Hence the more ideal operation, that which endeavours to restore most perfectly the *status quo ante*, is also by far the safest for the patient. Hutchinson and Barker, in discussing[3] these propositions, were in favour of performing the resection, not at the site of the hernia, but through a second opening in the middle line. Franks prefers immediate suture of the divided ends of the intestine by means of Gély's plan—a form of suture which picks up the peritoneal coat only. It is passed in the following manner: A long fine piece of silk is armed at each end with a fine needle. One of these needles is passed through the peritoneal coat of the upper end of the divided intestine, about a quarter of an inch from the edge, and, passing parallel to the divided edge beneath the peritoneum, emerges again at a point about a quarter of an inch from the point at which it entered. The second needle enters the peritoneum covering the lower end of the divided gut at a point exactly opposite to the point of entrance of the first needle, and emerges at a point exactly corresponding to the point of emergence of the first needle. When the silk is drawn tight the edges of the intestine invert, and when accurately coapted the suture is tied in a knot. The needles are then passed in again, starting from the knot, and each needle picks up another quarter of an inch of peritoneum on each side, and the suture is again tied. This process is repeated until the whole circumference of the bowel has been dealt with. It will thus be seen that the suture is a continuous one, but interrupted at every point of emergence and entrance by a knot. Franks reports[4] three cases of enterectomy in which the divided intestine was united in this way. Mr. Treves has also published[5] a case of reunion of the colon by simple suture after exsection, in which the divided ends were united by Lembert's sutures—about fifty stitches being employed.

Intestinal anastomosis by means of Senn's plates is no doubt a rapid method of approximating portions of divided intestine of very unequal calibre, but there has been for some time a growing feeling that the use of these decalcified plates is not altogether satisfactory, and the subject of intestinal anastomosis has therefore received much attention lately from experimenters in this branch of abdominal surgery. The disadvantages of the boneplates of Senn are: (1) Danger of invagination; (2) the plates pressing on the lumen of the bowel, causing gangrene; (3) the final fate of the plates is not always certain; (4) the opening is apt to become occluded.

In experiments by Thomson[6] the following technique was carried out. After resection of the small intestine, a fine needle with a feather eye, armed with a long silk thread, is carried through the bowel from the mucous side one millimetre from the line of incision. It is then pushed through the other end of the bowel at a corresponding point from the serous to the mucous side. The suture is left long, and the needle again introduced in the same manner from two to three millimetres from the original puncture, and so on around the entire circumference. The sutures are then knotted, the latter all being in the lumen of the bowel. The serous surfaces are well approximated. The knots should not be drawn too tight for fear of causing necrosis. Mr. Stanley Boyd operated successfully on a case of artificial anus of six months' duration, left after an operation for strangulated hernia. He pared and united the ends of the bowel by Maunsell's method, and expresses[7] his opinion that Maunsell's method of enterorraphy is the simplest of the methods before the profession, no special apparatus being required: that it is as quick as, if not quicker than, any of them, and that there is sufficient evidence in favour of its safety to warrant a further trial. McGraw, too, in view of the frequent recurrence of stenosis after the various operations for the production of anastomosis of the hollow viscera, describes[8] his method, which aims at making permanent openings not liable to contraction. In most operations hitherto employed the intention is to produce adhesion of the serous surfaces alone, and a wide granulating edge, uncovered by mucous membrane, bounds the entire circumference of the orifice, hence contraction occurs. Some have endeavoured to obviate this by making very large openings. The principle on which McGraw's operation is based is the same as that which is made use of in operating for webbed fingers, namely, to break the line of scar by the interposition of sound integument or mucous membrane. Incisions are made in the viscera it is intended to join by which flaps are formed and turned back and stitched, thus making an opening the edge of which is wholly or in part, depending on the form of incision, lined by everted mucous membrane. If it is arranged to have one raw edge, this would come opposite to the flap in the other viscus, and be fastened to the serous surface just outside the everted flap. Other observers, however, have been endeavouring to simplify and accelerate the operation of intestinal anastomosis by mechanical appliances, which they believe to be quicker than by immediate suture, and without the disadvantages attending union by means of Senn's plates. Dr. Ramaugé has devised[9] a proceeding for uniting the ends of severed intestine without suturing, to which he has given the name of enteroplexy. This he effects by means of a little instrument of his invention, which he calls the enteroplex. It is a kind of clamp, consisting of two smooth

Murphy's Button.—The cylinder of the lower half passes into that o the upper. The two teeth, projecting through holes in the lower cylinder catch on a thread inside the upper cylinder so that once the two parts are pushed together they can only be separated by unscrewing them.

aluminium rings, over the opposite ends of which the severed extremities of the intestine are infolded and secured with a few sutures; then the rings—having between them the double thickness of infolded intestine—are pressed together, and two small springs fix them in this position. The instrument is subsequently shed into the bowel, and voided per anum. To test its efficiency Dr. Ramaugé has experimented on pigs. In a case in which the small intestine had been transversely divided, and then secured with the instrument, it was found when the animal was killed twenty-one days later that the gut had perfectly united without any bad symptoms. On another animal ileo-ileostomy was thus done. Perfect adhesion was found to have taken place when the animal was killed fourteen days later.

This operation is similar to the better-known device for intestinal anastomosis without suture, which Murphy calls[10] an "anastomosis button." It consists of two small circular bowls made of metal. In one bowl is fixed a small cylinder with a female screw thread on its entire inner surface. In the other bowl is fitted a smaller cylinder of a size to slip easily into the female cylinder. On the inner surface of the lower end of the male cylinder two small brass springs are fixed, which serve to hold the bowls together when the male cylinder is pressed into the female cylinder. In the side of each bowl are four openings which serve for drainage. The whole button has the form of two hemispherical bodies held together by invaginating cylinders. These hemispheres of the button are inserted in slits or ends of the viscera to be operated upon. A running thread is placed around the slit in the viscus, so that when it is tied it will draw the cut edges within the clasp of the bowl. A similar running thread is applied to the slit in the viscus, into which the other half of the button is inserted, and the bowls are then pressed together. The pressure atrophy at the edge of the bowl is produced by a brass ring supported by the spring. The opening left after the button has liberated itself is the size of the button. For this device the inventor claims the following advantages: (1) It retains its position automatically; (2) it is independent of sutures; (3) it produces a pressure atrophy and adhesion of surfaces at the line of atrophy; (4) it ensures a perfect apposition of surfaces without the danger of displacement; (5) it is applicable to the lateral as well as to the end-to-end approximation; (6) it produces a linear cicatrix, and thus ensures a minimum of contraction; and (7) it is extremely simple in its technique. Murphy has recorded three cholecyst-enterostomies and four gastro-enterostomies in the human subject. All were successful. In one case the time occupied by the operation was eleven minutes. Other experimental operations on animals are reported as having been done successfully. These include cholecyst-enterostomy, gastro-intestinal anastomosis, and entero-intestinal anastomosis. Mr. Keen has published[11] the first post-mortem recorded after the use of Murphy's button for the purpose of ileo-colostomy, in a case which died about six weeks after the operation. At the point of anastomosis a circular aperture was seen, the union being perfect. It measured half an inch in diameter. The size of the button by which the opening was made was one inch in diameter, showing that the aperture, in the forty-seven days since the operation, had contracted to one-half of its original diameter. This, Mr. Keen thinks, is an important point and suggests the query whether the contraction would not have gone on until it practically would have rendered the anastomosis fruitless, thus fulfilling the fear expressed by Dawbarn.[12] In gastro-enterostomy the contraction would be a serious matter after a time, and might make the operation of no avail. If, also, the button should drop back into the stomach, or into the proximal cul-de-sac of the bowel in a lateral anastomosis, it would never be able to escape through the constantly narrowing orifice. This has convinced Mr. Keen that the button should be abandoned for intestinal or gastro-intestinal anastomosis. It will find, however, a most useful field in cholecyst-enterostomy, where only bile and no solid matter has to pass through the opening, and the contraction would not be an obstacle to permanent success.

Mayo Robson now accomplishes intestinal anastomosis by means of decalcified bone tubes, shaped like a cotton bobbin. The advantages he claims[13] for his method are: (1) Rapidity of execution; (2) simplicity and ease of performance, only two continuous sutures being required; (3) the avoidance of leaving large plates in the intestine; (4) security against leakage by the double continuous suture; (5) the certainty of having an adequate and immediately patent opening; (6) the avoidance of the danger of after-closure of the opening by securing continuity of mucous surfaces throughout the new channel; (7) the adaptability of the principle to (*a*) lateral intestinal anastomosis, (*b*) lateral implantation as in ileo-colostomy, (*c*) gastro-enterostomy, (*d*) pylorectomy, (*e*) end-to-end enterorraphy after enterectomy, and (*f*) cholecyst-enterostomy.

The operation is thus performed. For the sake of example let it be supposed that it is required to connect the ileum to the sigmoid flexure of the colon. An incision is made in the abdominal parietes in the usual position for inguinal colotomy, and a loop of the sigmoid brought up and drawn between the fingers of the left hand, so as to empty it of blood and intestinal contents, and then clamped. In the same way a loop of ileum is emptied and clamped, both loops being brought outside the abdomen. Nibbed forceps are applied as guides to the ileum and colon where they are to be opened. Two 18-inch long continuous sutures, threaded on curved sewing needles, are ready; the outer, of silk, is applied half an inch from the place where the viscera are to be opened, first to ileum and then to colon alternately, the suture taking up peritoneum and muscular coat only, and each suture taking up about one-third of an inch of surface. The suture is commenced on the right, proceeding to the left, the tail end of the suture remaining long, and

Fig. 1.—Showing serous suture applied around the posterior half circle.

when the extreme left is reached the needle is not unthreaded, in order to complete the suturing after the bone-tube has been inserted and the marginal suture completed. The viscera are then incised, the opening being just sufficient to admit the bone-tube; but before its insertion, the marginal suture, which may either be of chromicised catgut or of silk stained with aniline, is applied from right to left, uniting the posterior margins of the two visceral openings, the suture including

Fig. 2.—Showing the marginal suture applied around the posterior half circle.

mucous membrane, the tail of the suture being left long on the right and being kept threaded on the left. The tube is now inserted, one end being in the ileum, the other in the colon.

Fig. 3.—Showing tube in position, and the anterior part of the marginal suture continued around the circle.

The marginal suture is then proceeded with around the front until the tail of the suture is reached; the two ends are then drawn tight, tied, and cut off short, thus uniting the mucous surfaces around the tube. The outer serous suture is then proceeded with half an inch from the marginal suture until the circuit is completed, when the two ends are drawn upon, tied, and cut off short. The sutures cannot then be seen, and the anastomosis is complete.

Robson reports cases of ileo-colostomy and gastro-

Fig. 4.—Diagram to show shape and measurements of tube.

enterostomy performed by this method. The accompanying diagrams show the form and size of the bobbins. The bobbins, however, vary in size, and the tube employed for cholecyst-enterostomy has its barrel only as thick as a No. 16 English catheter. In the performance of operations on the intestines, some operators prefer to use, while others discard, an intestinal clamp, but such instruments are certainly of use when a reliable assistant is not at hand. The latest of these clamps for enterectomy is that of Dr. James Swain,[14] in which an equality of pressure is maintained along the whole length of the blades, by applying the power at the centre of the blades, which swing on short

Dr. James Swain's Clamp for Enterectomy.

secondary shanks, placed at right angles to the main shanks. By these means the pressure remains uniform, whether the blades be parallel or not, and thus ensures that the intestine is not unduly compressed in any one part.

[1] Dub'in Med. Journ., June, 1893. [2] Med. Rec., vol. xliii., No. 14. 1893. [3] Brit. Med. Journ., No. 1,683, 1893. [4] Op. Cit. [5] Lancet, March 11, 1893. [6] Zeitschrift fur Geburt, und Gynœkol, Band 26. [7] Lancet, May 27, 1893, p. 1,281. [8] Annals of Surgery, Sept., 1893, vol. xviii., No. 3, and Brit. Med. Journ., Oct. 7, 1893. [9] Med. Chron., Oct., 1893. [10] Med. Record, Dec. 10, 1892. [11] Annals of Surgery, June, 1893. [12] Annals of Surgery, Feb., 1893, p. 155. [13] Brit. Med. Journ., April 1, 1893. [14] Brit. Med. Journ., Dec. 16, 1893.

## Figures and Tables

**Figure f1:**
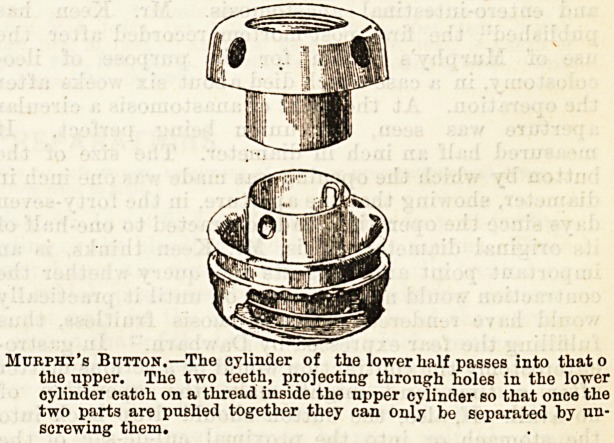


**Fig. 1. f2:**
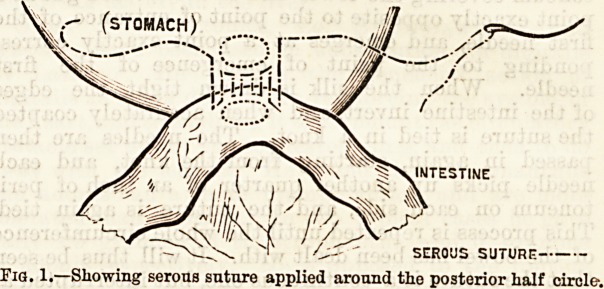


**Fig. 2. f3:**
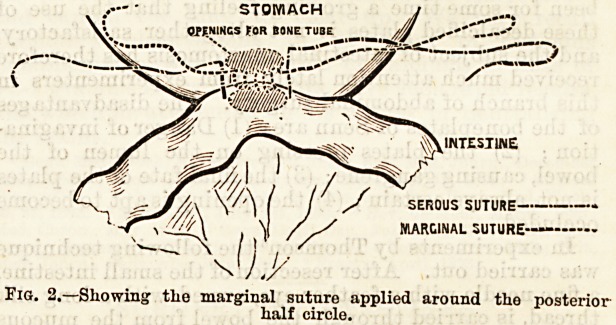


**Fig. 3. f4:**
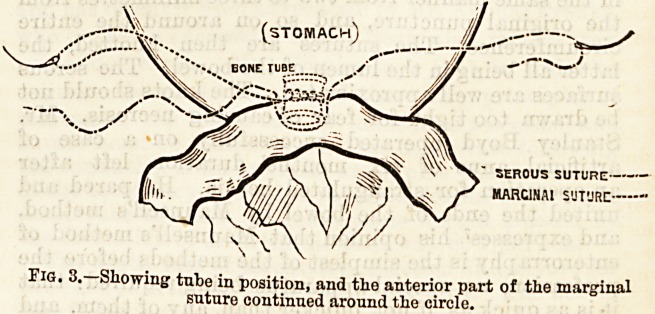


**Fig. 4. f5:**
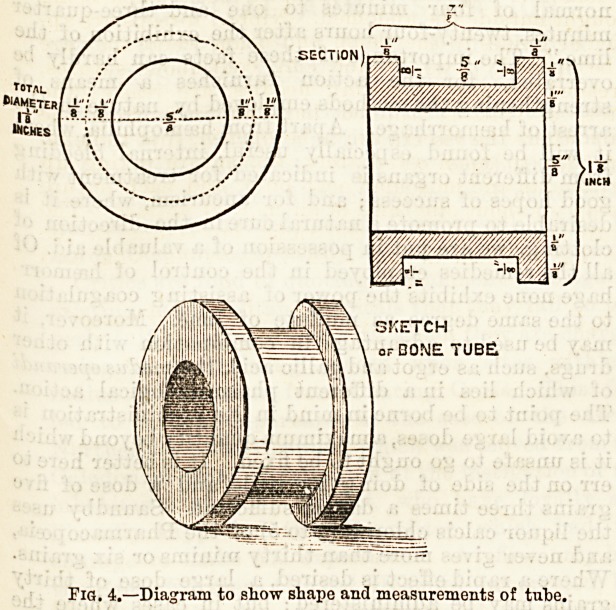


**Figure f6:**